# Positive selection of skeleton-related genes during duck domestication revealed by whole genome sequencing

**DOI:** 10.1186/s12862-021-01894-7

**Published:** 2021-09-06

**Authors:** Tao Zhu, Xin Qi, Yu Chen, Liang Wang, Xueze Lv, Weifang Yang, Jianwei Zhang, Kaiyang Li, Zhonghua Ning, Zhihua Jiang, Lujiang Qu

**Affiliations:** 1 Department of Animal Genetics and Breeding, National Engineering Laboratory for Animal Breeding, College of Animal Science and Technology, China Agricultural University, Yuanmingyuan West Road 2#, Beijing, 100193 China; 2 Beijing General Station of Animal Husbandry, Beiyuan Road 15A#, Beijing, 100107 China; 3 Department of Animal Sciences, Center for Reproductive Biology, Veterinary and Biomedical Research Building, Washington State University, Pullman, Washington 647010 USA

**Keywords:** Duck, Domestication, Artificial selection, Positive selection, Skeleton

## Abstract

**Background:**

Domestication alters several phenotypic, neurological, and physiological traits in domestic animals compared to those in their wild ancestors. Domestic ducks originated from mallards, and some studies have shown that spot-billed ducks may have also made minor genetic contributions to domestication. Compared with the two ancestral species, domestic ducks generally differ in body size and bone morphology. In this study, we performed both genomic and transcriptomic analyses to identify candidate genes for elucidating the genetic mechanisms underlying phenotypic variation.

**Methods:**

In this study, the duck genome data from eight domestic breeds and two wild species were collected to study the genetic changes during domestication. And the transcriptome data of different tissues from wild ducks and seven domestic ducks were used to reveal the expression difference between wild and domestic ducks.

**Results:**

Using fixation index (Fst) algorithm and transcriptome data, we found that the genes related to skeletal development had high Fst values in wild and domestic breeds, and the differentially expressed genes were mainly enriched in the ossification pathway. Our data strongly suggest that the skeletal systems of domestic ducks were changed to adapt to artificial selection for larger sizes. In addition, by combining the genome and transcriptome data, we found that some Fst candidate genes exhibited different expression patterns, and these genes were found to be involved in digestive, immune, and metabolic functions.

**Conclusions:**

A wide range of phenotypic differences exists between domestic and wild ducks. Through both genome and transcriptome analyses, we found that genes related to the skeletal system in domestic ducks were strongly selected. Our findings provide new insight into duck domestication and selection effects during the domestication.

**Supplementary Information:**

The online version contains supplementary material available at 10.1186/s12862-021-01894-7.

## Background

Domestication is the process in which a wild species is artificially selected to be domestic, with many traits changed to meet human requirements. Various features distinguish domestic animals from their wild ancestors, and it is well-documented that the genetic architecture underlying variations in phenotypic traits (such as morphology in chickens [1, 2], pigs [3–5], and pigeons [6, 7]; feather color [8, 9]; neurological aspects such as tameness in companion animals [10–12]; and physiological aspects such as starch digestibility in dogs [13] and reproduction in chickens [14, 15]) are altered dramatically in domestic animals compared to those in their wild ancestors.

Archeological evidence shows that China has a long history in ducks domestication, the origin of domestic ducks is controversial. A previous study showed that Chinese domestic ducks mainly originated from mallard (*Anas platyrhynchos*) and may have been introgressed by spot-billed ducks (*Anas zonorhyncha*) [16]. Compared to wild ducks, domestic ducks do not require evasion from predators and foraging, causing domestic ducks to retain their various plumage colors [9], such as white in Pekin ducks. Furthermore, wild ducks maintain their flight capability for migration, whereas domestic ducks are flightless. The domestication process has greatly changed the body shape, including the size, elevation, and fat content. These phenotypic differences indicate that large changes have occurred in the genome, and is important for animal genetics to identify the genes selected during the domestication process.

In this study, two wild duck species and eight domestic duck breeds were selected to investigate the potential selected genes in domesticated ducks using genomic and transcriptomic data. We found that some genes associated with bone development were positively selected and differed at both the genomic and transcriptomic levels between wild and domestic ducks.

## Methods

### Sampling and genomic and transcriptomic sequencing

The genome sequencing data of two ancestral wild populations and eight domestic breeds were used in this study. The ancestral wild duck including mallard (MD, n = 21) and spot-billed ducks (SB), domesticated populations including Pekin duck (PK, n = 8), Cherry Valley duck (CV, n = 8), Maple Leaf duck (ML, n = 8), Jinding duck (JD, n = 8), Shaoxing duck (SX, n = 8), Mei duck (Mei, n = 8), Shanma duck (SM, n = 8), and Gaoyou duck (GY, n = 8). Mei was obtained from Anhui Province, China, SB was collected from Ningxia Province, China. The remaining ducks were obtained from our previous study [8]. Blood samples were collected from the brachial veins by standard venipuncture, and all the ducks were released. Genomic DNA was extracted from newly collected samples using the standard phenol/chloroform extraction method, and two paired-end libraries (500 bp) were constructed using the Illumina HiSeq 2500 sequencing platform (San Diego, CA, USA). In addition, the liver, brain, and breast muscle tissues from 14 adult male ducks (MD, n = 7; PK, n = 1; CV, n = 1; ML, n = 1; JD, n = 1; SM, n = 1; SX, n = 1; GY, n = 1) were collected for RNA sequencing (RNA-seq). The detailed sample information and sequencing methods have been described in our previous study [8].

### Pretreatment of genomic data

The raw genome data were first processed using fastp (v0.20.0) to filter adapter contamination and low-quantity reads [17]. The clean data were aligned to the duck genome (https://www.ncbi.nlm.nih.gov/, accession: GCF_003850225.1) using BWA-MEM (v0.7.15) [18]. Picard (http://broadinstitute.github.io/picard/) was used to sort the aligned data and remove duplicate reads. Genome Analysis Toolkit v3.6 (https://gatk.broadinstitute.org/) was used for variant calling and variant filtering. SNPs were filtered using the following rules: (a) QUAL > 30.0, (b) QD > 5.0, (c) FS < 60.0, (d) MQ > 40.0, (e) MQRankSum > − 12.5, and (f) ReadPosRankSum > − 8.0. Additionally, if there were more than three SNPs clustered in a 10-bp window, they were all considered as false positives and removed [19]. Finally, credible SNPs in VCF format were retained for subsequent analysis.

### Population structure and phylogenetic trees

The SNP file was converted to plink format using VCFtools (v0.1.13) [20], and low-quality SNPs with a missing rate greater than 0.1 [21]. Principal component analysis was conducted with plink [22], and the top 20 components were used for population relationship analysis. To construct the phylogenetic tree, SNPs that overlapped with the CDS were extracted, and the maximum likelihood phylogenetic tree was constructed using iqtree2 with the GTR + ASC model [23]. iTOl was used to visualize the phylogenetic trees [24].

### Selective-sweep analysis

To define candidate direct selection regions, the wild ducks (MD and SB) were placed in one group, and the remaining ducks were clustered into another group. The Fst was calculated with 50-kb windows and slides with 25-kb steps; genome regions overlapping with upstream and downstream 50 kb of the top 1% Fst windows were considered as candidate regions, and genes with overlapping candidate regions were considered as candidate genes.

### RNA-seq data analysis

Raw transcriptome data were processed using fastp (v0.20.0) [17], and the clean data were aligned to the duck genome (https://www.ncbi.nlm.nih.gov/, accession: GCF_003850225.1) using HISAT2 (v 2.1.0) [25]. The aligned files were sorted using SAMtools (v 1.9) [26]. The reads number for each gene was counted using featureCounts (1.6.4) [27]. The reads count files were merged with tissue, and expression levels were calculated using DESeq2 [28]. Genes with an absolute value of expression level change greater than 1.5-fold and adjusted *p* value less than 0.05 were considered as DEGs. Metascape was used for gene enrichment analysis [17].

## Results

### Population structure

**Fig. 1:**
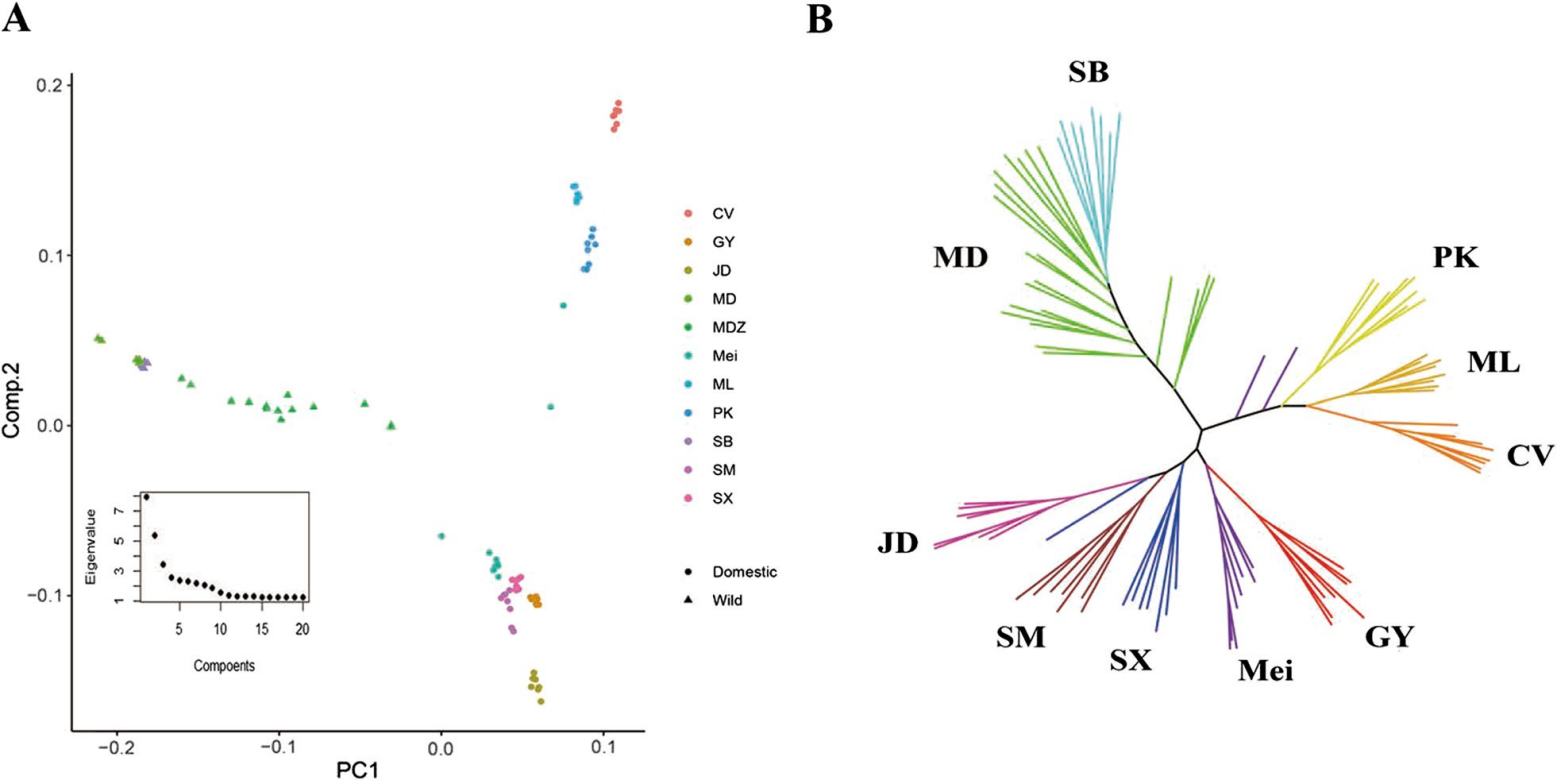
Population structure of ducks. **A** Principal component analysis plot of ducks. The inner dot plot represents the eigenvalues of the top 20 principal components. **B** Maximum likelihood phylogenetic tree of all ducks

From the genome data, we obtained 38,097,848 high-quality single-nucleotide polymorphisms (SNPs) with an average depth of 564. To study the population structure, we calculated the top 20 components of the experimental ducks; the top three components explained 37.68% of the total variance (Fig. 1A). The results showed that the wild and domesticated ducks were classified by a line in which component 1 equals 0, whereas component 1 of wild ducks was less than 0 (Fig. 1A). All individuals were clustered into three groups, with the wild species MD and SB separated from PK, CV, and ML ducks, which were clustered into one group, and the remaining ducks clustered together in another group. To study the phylogenetic relationships among the ducks, coding sequence (CDS) SNPs were used to construct the maximum likelihood evolutionary tree, which showed that SB and MD were clustered into one branch and PK, CV, and ML were clustered into another branch, and the remaining breeds were grouped together (Fig. 1B).

### Skeletal system development genes were positively selected during domestication

**Fig. 2:**
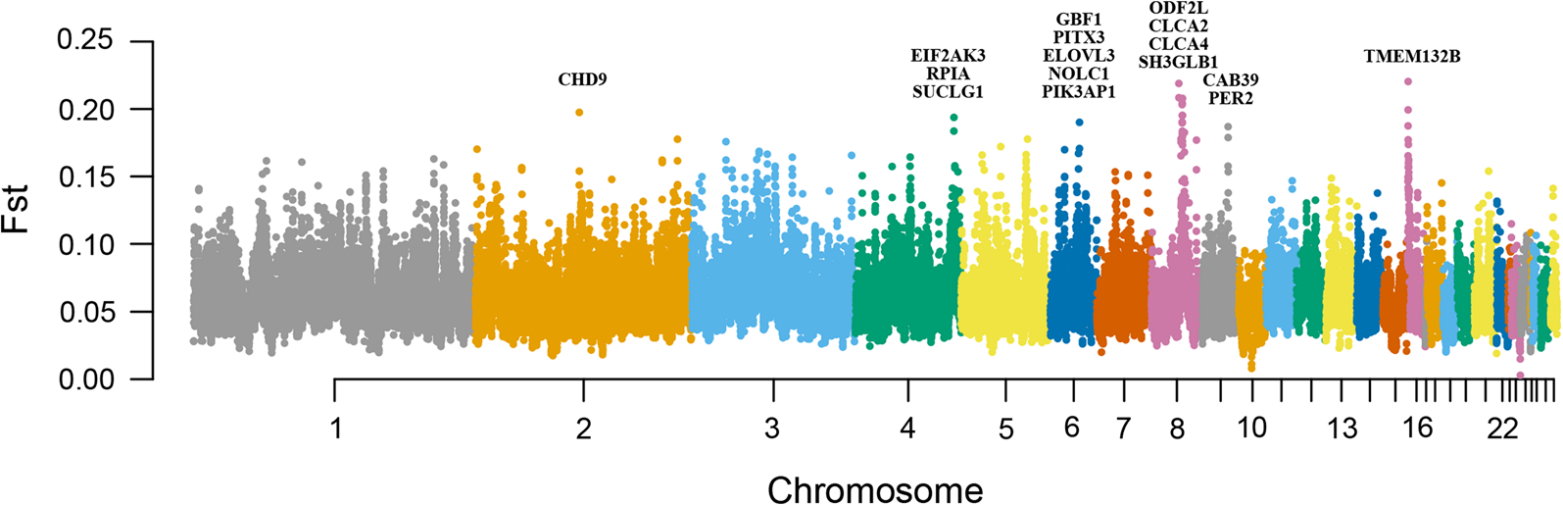
Manhattan plot for Fst and genes that overlap with sweep peaks

**Fig. 3:**
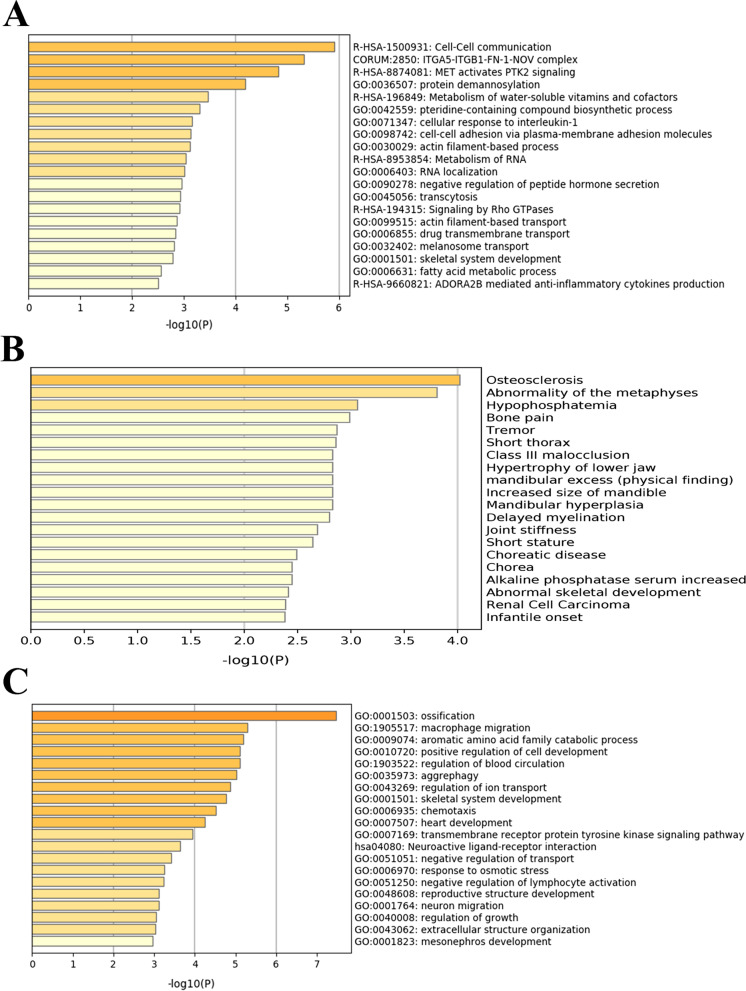
Gene enrichment analysis for candidate genes. **A** Gene Ontology (GO) analysis for Fst candidate genes. **B** DisGeNET enrichment result for Fst candidate genes. **C** GO analysis for DEG in the breast muscle

The slide window fixation index (Fst) algorithm was employed to identify candidate genes that diverged between wild and domesticated ducks. To avoid bias caused by sex chromosomes, only autosomes were further analyzed. We obtained 39,251 autosome windows, with a mean Fst of 0.061 between wild and domesticated ducks. The two peaks of chromosomes 16 and 8 exceeded 0.2. The maximum Fst value was 0.22 at chromosome 16:300,001 bp–350,001 bp, which overlapped with the TMEM132B gene, and the second peak at chromosome 8:19,750,001 bp-19,800,001 bp, overlapping with the LOC101797367 gene, also known as CLAC4 (Fig. 2). We extracted genes overlapping with the top 1% window or adjacent windows as candidate genes. Finally, we identified 525 candidate genes and 260 genes mapped to the human genome. Metascape was used to annotate the gene functions [29]. Many gene ontology (GO) items were related to metabolism and cellular energy conversion (Fig. 3A). One GO item, GO:0001501, was significantly correlated with skeletal system development, and 14 genes were included in this item. Enrichment analysis on the DisGeNET platform showed that the candidate genes were related to bone diseases, such as osteosclerosis, bone pain, and abnormality of the metaphyses (Fig. 3B) [30]. In addition, the EIF2AK3 gene on chromosome 4 overlapped with the third highest peak. This gene was associated with body size, abnormal skeletal morphology, and abnormal hind limb morphology in mice [31, 32].

### Differentially expressed genes (DEGs) in muscle predominantly enriched in bone developmental pathway

Gene function can be determined based on the RNA transcription level at a specific time. The breast muscle, liver, and brain from wild and domesticated male ducks were collected for gene expression analysis. The expression level of each gene was standardized using the FPKM algorithm [33], and DEGs were identified by their fold-change and adjusted *p* value. After filtration, 112, 31, and 180 genes in the liver, brain, and breast muscle, respectively, were identified as DEGs. The number of DEGs in the breast muscle was significantly larger than that in the brain and liver. GO analysis was used for gene function analysis. DEGs in the breast muscle were enriched in the ossification and bone development pathways (Fig. 3C). DEGs in the liver were enriched in the muscle organ development and cartilage development pathways (Additional file 1: Figure S1A). The brain was enriched in the visual perception and hormone regulation pathways (Additional file 1: Figure S1B).

Next, we extracted the overlapping genes between the Fst and DEGs analyses, and identified 6, 2, and 10 genes in the liver, brain, and breast muscle, respectively, showing different expression patterns and high Fst values (Table 1). These genes are involved in the immune, digestive, and metabolic systems. The most divergent gene, TMEM132B, was highly expressed in the wild duck breast muscle.

**Table 1:** Differentially expressed candidate genes in the breast, liver, and brain

Breast	Liver	Brain
HHLA2 (immune)	LOC113842605	LOC113844317
IFT57 (body development)	LOC106019145	LOC106015673
LOC101795816 (immune)	NMNAT2 (metabolism)	
LOC106019145	LAMC2 (body development)	
LOC101798418 (metabolism)	LOC101794647 (metabolism)	
SGMS2 (bone)	LOC113844272 (metabolism)	
DDX60 (immune)	GXYLT2 (metabolism)	
LOC101800182 (metabolism)		
LOC110351937		
LOC106017289		

Terms in parentheses represent the gene functional classification

## Discussion

Domestication, including captivity and artificial selection, has resulted in large morphological changes in ducks. Breeding has also introduced a large number of variants into the domesticated duck genome. In our study, wild and domesticated ducks were distinguished by the first principal component (Fig. 1A), which showed that long-term artificial selection led to tremendous variation in the duck genomes. In previous studies, the differences were mainly reflected in agronomic traits, such as egg production, growth rate, and fat deposition [8, 34]. Domestic ducks were clustered into two groups by the second principal component, corresponding to their biological origin. Our results suggest that genetic distances in Chinese indigenous ducks are related to geographical distances, and indigenous ducks in southern China may have originated from a common ancestor. With the development of sequencing technology, phylogenetic analyses of ducks have been performed at the whole-genome scale. These studies demonstrated that mallards are the ancestors of domestic ducks [8, 9], and Liu et al. [35] found that MD and SB specific SNPs were equally distributed into eight domestic ducks. Taken together, both MD and SB ducks are thought to have contributed to domestic ducks.

In our study, the Fst algorithm was used to identify divergent genes. In total, 525 genes were identified as candidate divergent genes. One GO item containing 14 genes was related to bone development, and the DisGeNET database showed that our candidate genes were related to bone diseases. Furthermore, bone-related DEGs in the breast muscle showed different expression patterns, suggesting that the musculoskeletal system changed dramatically during duck domestication. These changes may be attributed to the limited activity space during domestication. In chickens, bone mineral density, trabecular bone volume, and trabecular microarchitecture were found to be significantly reduced when chickens were kept in small cages [36]. Therefore, domestic ducks may have lower bone strength and density. In our study, we found that the EIF2AK3 gene greatly differed between domestic and wild ducks, and many studies showed that mutations in the EIF2AK3 gene can lead to Wolcott-Rallison syndrome [37–39], a rare autosomal recessive disorder characterized by permanent insulin-dependent diabetes, multiple epiphyseal dysplasia, and growth retardation. In mice, EIF2AK3 is associated with the hindlimb morphology and body weight [31, 32, 40]. The EIF2AK3 gene plays an important role in the development and morphology of the domestic duck bone. This functional change in bone development may have led to the loss of flight in domestic ducks. Compared to their wild relatives, wild birds have a lighter mass, larger wing area, and greater bone length [41–43]. These differences are consistent with the major breeding goals. For example, as one of the most famous meat-type ducks worldwide, PK were bred for their fast growth rate and high fat deposition; additionally, their body weight can reach up to 3.1 kg at 35 days of age, whereas the adult MD weighs only 1.1–1.2 kg [44–47]. Although breeding greatly increased the body weight of ducks, long-term captivity weakened the importance of wings, which was accompanied by physiological changes such as flight muscle atrophy and decreased bone strength and wing size (limbs). For example, in a flightless steam duck (*Tachyeres patachonicus*), wing loading, humerus length, radius length, and ulna length were significantly changed compared to those in their flying relatives [41].

TMEM132B and CLCA4 showed the highest Fst values. CLCA4 is involved in regulating electrolytic fluxes and thereby modulates secretion, absorption, cell volume, and membrane potential. CLCA4 was also found to be highly expressed in the human colon and esophagus, and variants of CLCA4 were associated with cystic fibrosis [48, 49], an inherited disorder that severely damages the lungs and digestive system. Selection of the CLCA4 gene may be related to changes in food composition. The TMEM132B is a member of the TMEM132 gene family [50]. The TMEM132 family is composed of neuronal genes, and mutations in the TMEM132 gene can cause nervous system diseases such as insomnia symptoms and hearing loss [51, 52]. A similar study in cattle showed that TMEM132E was positively selected [53]. Transcriptomic analysis revealed that some Fst candidate genes, mainly those with immune and metabolic functions, had different expression patterns. Interestingly, two genes (LOC101794647 and LOC113844272, also known as pancreatic alpha-amylase and pancreatic alpha-amylase-like, respectively) were found to be related to starch digestion, possibly because of the grain present in domestic duck feed. A similar selection effect was found in dogs, in which the starch digestion gene MGAM was selected and highly expressed [13]. In addition to bone development, our data suggest that the digestive, nervous, and immune systems were impacted during selection for breeding.

## Conclusions

By comparing two wild duck species with domesticated duck breeds using both genomic and transcriptomic data, we found that domestic ducks changed in many aspects during domestication, particularly in the skeletal system.

## Supplementary Information

**Additional file 1: Figure S1.** Gene Ontology (GO) analysis of differentially expressed genes in the liver (A) and brain (B)

## Data Availability

The data supporting the conclusions of this article are available from NCBI (https://www.ncbi.nlm.nih.gov/, PRJNA686828).

## Abbreviations

MD: Mallard
SB: Spot-billed duck
PK: Pekin duck
CV: Cherry Valley duck
ML: Maple Leaf duck
JD: Jinding duck
SX: Shaoxing duck
Mei: Mei duck
SM: Shanma duck
GY: Gaoyou duck
SNPs: Single-nucleotide polymorphisms
Fst: Fixation index
DEGs: Differentially expressed genes
CDS: Coding sequence
