# Supplementary material for: Positive selection of skeleton-related genes during duck domestication revealed by whole genome sequencing
Source: BMC Ecol Evol. 2021 Sep 6;21:165. doi: 10.1186/s12862-021-01894-7 (PMC8419914; doi:10.1186/s12862-021-01894-7)
Supplement: Supplementary file 1 — Additional file 1: Figure S1. Gene Ontology (GO) analysis of differentially expressed genes in the liver (A) and brain (B) [file 12862_2021_1894_MOESM1_ESM.pdf]

A

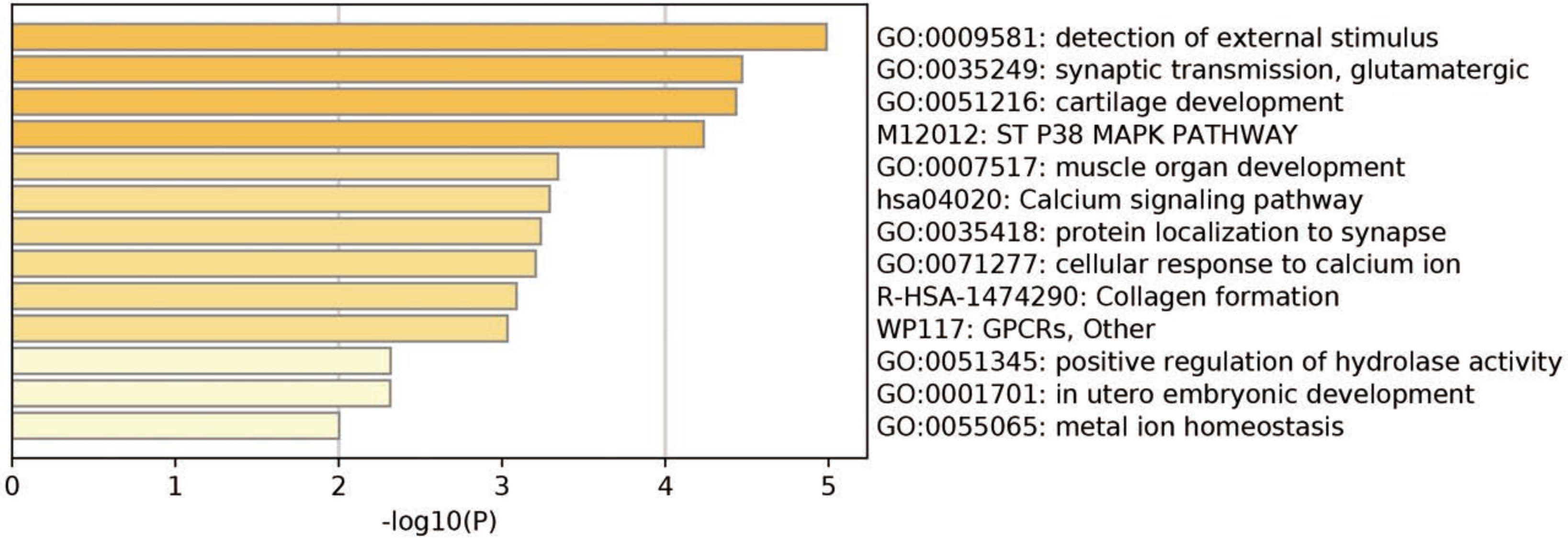

GO:0009581: detection of external stimulus
GO:0035249: synaptic transmission, glutamatergic
GO:0051216: cartilage development
M12012: ST P38 MAPK PATHWAY
GO:0007517: muscle organ development
hsa04020: Calcium signaling pathway
GO:0035418: protein localization to synapse
GO:0071277: cellular response to calcium ion
R-HSA-1474290: Collagen formation
WP117: GPCRs, Other
GO:0051345: positive regulation of hydrolase activity
GO:0001701: in utero embryonic development
GO:0055065: metal ion homeostasis
0
1
2
3
4
5
-log10(P)

B

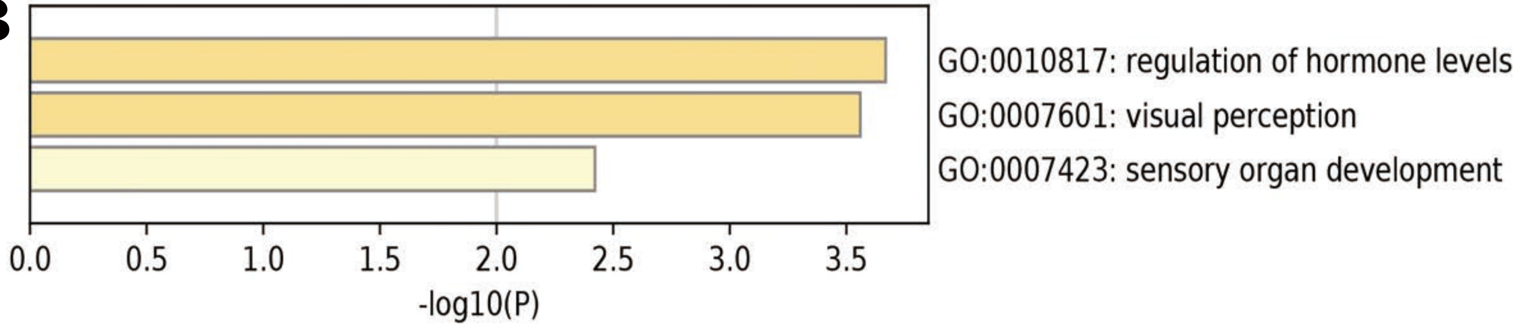

GO:0010817: regulation of hormone levels
GO:0007601: visual perception
GO:0007423: sensory organ development
0.0
0.5
1.0
1.5
2.0
2.5
3.0
3.5
-log10(P)
